# Editorial: Performance Analysis in Sport

**DOI:** 10.3389/fpsyg.2020.611634

**Published:** 2020-10-30

**Authors:** Miguel-Angel Gomez-Ruano, Sergio José Ibáñez, Anthony S. Leicht

**Affiliations:** ^1^Faculty of Physical Activities and Sport Sciences, Polytechnic University of Madrid, Madrid, Spain; ^2^Faculty of Sport Sciences, University of Extremadura, Badajoz, Spain; ^3^Sport and Exercise Science, James Cook University, Townsville, QLD, Australia

**Keywords:** performance, sport, notational analysis, athlete, sport performance

Performance analysis is a sub-discipline of Sport Science research (Borms, 2008) that has attained great interest for many stakeholders (i.e., coaches, technical staff, performance analysts, managers, media, fans, and players) at different levels of performance (i.e., youth, semi-professional, or professional players). The development and implementation of new technologies to measure individual or team's performances (e.g., tracking systems such as local positioning systems, LPS; video tracking, or observational video analysis systems) with multiple practical applications have intensified the focus of performance analysis in sport (Hughes and Franks, 2007). Traditional approaches have included static analysis focused on retrospective performances; however, dynamic and complex analyses (i.e., non-linear Multi-Dimensional Scaling, classification and regression tree, logistic regression, etc.) have become increasingly utilized by researchers for a deeper understanding of sport performance during training and competition (O'Donoghue, 2009). In particular, a holistic and multidisciplinary perspective such as the Grand Unified Theory analyses (GUT, see Glazier, 2017) has been suggested to be fundamental for sports performance. This approach, provides a framework to examine the inter- and intra-athlete's behavior dimensions under the environmental and task-related (ecological) factors that affect the performance. Specifically, isolated approaches have been suggested to be avoided with the integration of the biomechanical, physiological, psychological, technical, tactical, positional, motor development and/or strength and conditioning perspectives recommended when evaluating match-related contexts and training tasks (Glazier, 2017). Additionally, Woods et al. (2020) highlighted the importance of ecological dynamics to guide the control, preparation and assessment of athletes and teams. Subsequently, the use of interdisciplinary research designs would provide clear and well-described rationales, powerful data collection and analyses, resulting in robust findings. Innovative sports performance analyses that incorporate new technologies to understand individual's behaviors within real-based and ecological contexts would provide a greater understanding of how players and teams act and react for greater performance development and application (Bertollo et al., 2020). In fact, as Robertson (2020) argued, the development of professionalism and data gathering in sport had lead to a new scenario for coaching staff, athletes, and performance analysts where adaptative tools are essentially required to understand the needs of sports performance (e.g., human-machine interaction, perspective, innovation, versatility, visualization, evaluation, feedback, generalization, and future planning).

This special issue was initiated to gain a greater insight of current sport performance theory considering analytics and a wide range of sport disciplines (e.g., individual, dual, team sports) and variables studied (e.g., physical, positional, technical, tactical, psychological, or pedagogical). Of note was that this special issue drew immense attention resulting in the publication of 65 articles (and one corrigendum) improving the research knowledge of the following topics: performance profiling (18 articles), performance measurement (13 articles), reliability and validity of coding systems (eight articles), non-linear performances (seven articles), performances of females (four articles), tactical analysis (four articles), gender analysis (three articles), technical analysis (three articles), psychological aspects of performance (three articles), referees' performance (two articles), coaching issues (two articles), contextual variables (two articles), and new technologies in sport (two articles).

A focus on performance-related variables within sport has been the key issue studied for performance analysis. In particular, the use of fundamental indicators to model performance and establish performance profiling has provided the basis with high applicability for coaching staff to manage performance during training and competition. This approach was widely acknowledged in this Research Topic with 18 studies published. The analysis of soccer was presented in five studies from different perspectives and included the importance of technical indicators in the Big five teams of the UEFA Champions League (Yi, Groom et al.), running performances differentiated by Confederations (Tuo et al.), player's age and performance trends within the UEFA Champions League (Kalén et al.), and player migration and nationality (Lago-Peñas et al.). The analysis of other team sports showed the importance of multifactorial benchmarking and longitudinal performance in Australian football (McIntosh et al.), the long-term analysis of basketball players' performances (Lorenzo et al.), the multifactorial performance (physical, technical, and anthropometrical) of youth basketball players (Matulaitis et al.), the key determinants to succeed in wheelchair basketball [Francis et al.(a)], the positional, in-match running demands in rugby union (Donkin et al.), and the importance of ball type and innings in cricket batter's performance (Connor, Sinclair et al.). Individual sports were also investigated from different approaches including the relative age effect of track and field athletes (Brustio et al.), the world record profiles of long distance runners (Knechtle et al.), the spatiotemporal performance of 60 m hurdle athletes (González-Frutos et al.), the race strategies for long distance open-water swimmers (Veiga et al.), the longitudinal performance of elite rhythmic gymnasts (Sierra-Palmeiro et al.) and the pathway to succeed in elite swimming (Yustres et al.). Lastly, dual sports were studied with Torres-Luque, Fernández-García, Blanca-Torres et al. examining badminton (statistical analysis of men's/women's players), and Murray et al. examining squash (performance profiles of the top two players when considering the opponent's performance).

Measurement of performance analysis in sport was another main feature presented within this Research Topic with 13 articles covering different approaches and variables. Specific articles focused on postural skills and body related measures associated with sport expertise (Paillard), anthropometric measures (Eriksrud et al.; Gomez-Campos et al.), ranges of movement related to pain (Cejudo et al.), post-activation potentiation in soccer (Petisco et al.), and the effect of thermal conditions on performance (Gasparetto and Nesseler). Additionally, articles focused on physical fitness measures and performances in junior tennis players (Colomar et al.) and academy soccer players (Raya-González et al.) while physiological measures were considered when assessing elite male wheelchair basketball players' performance (Marszałek, Kosmol et al.). Finally, two studies investigated a research framework for the importance of several measures in Australian football (Bonney et al.) and the beginning of a senior career in team sports (Lupo et al.).

While the measurement of performance has been focused upon in this Research Topic, the reliability and validity of match-observation, as well as the observation systems used in performance analysis, were also extremely important with eight articles incorporated. In particular, the design and validation of observational instruments of technical and tactical actions were examined by Ortega-Toro et al. in soccer and Torres-Luque, Fernández-García, Cabello-Manrique et al. in tennis. A similar approach was undertaken by Francis et al. (b) who quantified actions in elite wheelchair basketball, and by Gong et al. who studied the validity of the CHAMPDAS match analysis system in elite soccer. Additionally, four studies considered reliability/validity procedures via various techniques; Belli et al. studied the reproducibility and validity of the stroke effectiveness in table tennis based on game temporal structure; Ibáñez et al. employed a learning and performance assessment instrument in basketball; Colino et al. validated an indoor tracking system that assessed activity distance and time for court-based sports; and Premelč et al. analyzed the reliability of judging artistic sport (danceSport).

A novel feature of this Research Topic was the coverage (seven studies) of the complexity and non-linear nature of sports that required different approaches to control for performance variability under unpredictable contexts. First, Ribeiro et al. focused their attention on bidirectional self-organizing tendencies in team sports with a specific approach based upon the game model and the principles of play. With this framework in mind, four studies analyzed soccer including: the interpersonal coordination perspective (i.e. importance of team dyads and task design) by Santos et al. with comments by Gesbert and Hauw in their commentary letter; the effect of interpersonal dynamics within 2 vs. 1 playing contexts (importance of field location and player roles) by Laakso et al.; and the effect of temporary numerical imbalances in youth players by Canton et al.. In addition, non-linear approaches were examined in beach volleyball ‘winning streaks' that considered the impact of failure in the temporal series (Link and Wenninger), and in padel which analyzed the effect of return of serve on the athlete movement patterns and rally outcome (Ramón-Llin et al.).

One of the most relevant and important issues in our collection of performance analysis, due to its high applicability, was tactical analysis with the following four studies: (i) Spencer et al. studied the quality of player's passing decisions in Australian football using commitment modeling that accounted for spatial influence and bounds/density of players; (ii) Méndez et al. analyzed the attacking patterns of elite futsal teams from Spain, Italy, and Russia that also considered the importance of efficiency, offensive organization, match type, scoring first and match outcome; (iii) Kim et al. investigated the attacking process in soccer from a goal scoring approach with the establishment of a taxonomy of how teams developed their attack when creating scoring opportunities; and (iv) Scharfen and Memmert analyzed the importance of cognitive function and specific-related motor skills during different tasks by elite youth soccer players.

A distinctive gender focus in performance analysis was also of importance within this Research Topic. Firstly, Pedersen et al. focused on the gender differences of soccer players based upon physiological and anthropometrical factors. Specifically, they presented a detailed approach to analyse and tailor-design training and competition based on gender differences. Secondly, Mclean et al. presented a work domain analysis that allowed the modeling of performance in women's netball. This research established the importance of complex relationships between key performance indicators, such as passing and possession measures, cognitive performance, and physical demands. Finally, two studies focused on the technical and tactical actions of elite soccer female players during the FIFA World Cup (Sainz de Baranda et al.) and the physical and external loads experienced by amateur women's basketball players (Reina et al.).

The importance of the technical analysis was also reflected in three studies with each focused on a different sport (i.e., cross-country, soccer, and cricket). Tjønnås et al. identified the basic motion patterns of cross-country skiing athletes and the need to control for physical, track, and environmental factors that influence these patterns. Additionally, Yi, Liu et al. studied the technical performance indicators of soccer players over nine seasons of the UEFA Champions league. Their results via Poisson regression and autocorrelation models showed trivial changes for shooting variables and defensive actions, but higher variability of passing and attacking-related variables. Lastly, Connor, Mann et al. studied the performance advantages of junior cricket batters based upon batting stance, lateral dominance, and type of technique. Their results highlighted the left-handed advantage and the need to control for these factors during team selection practices.

Two studies focused on the importance of contextual-related variables and their impact on players and teams' performance. Firstly, Pino-Ortega et al. analyzed the importance of the situational factors that effected external loads of Under-18 basketball players according to their playing position (i.e., team quality, match period, and consecutive matches). Secondly, Marszalek, Gryko et al. studied the heart rate profile of elite wheelchair basketball players who were classified according to their functional classification and playing time. Their results identified different performance trends according to the contextual factors of tournament level, game type, and game quarter.

The psychological approach in performance analysis was covered by three studies reflecting the importance of multifactorial analyses as key factors in sports performance. These articles focused on: analysis of self-control during pressure situations of penalty kicks in soccer (Navia et al.); the importance of competitive psychological disposition and self-perception of performance in youth female soccer players (Olmedilla et al.); and the psychological demands and well-being needs of Elite South African rugby players (Kruyt and Grobbelaar).

While a focus of the articles in this special issue was on players, the referee and coach were also examined. For referees, Kolbinger and Stöckl analyzed two common rule violations that were rarely penalized (i.e., misbehavior during penalty kicks and the goalkeeper holding the ball for more than 6 s) with their results from the German Bundesliga indicating that when players committed offenses, the referee's accuracy was only 20.8% for these situations. Likewise, Kraak et al. studied the rate of sanctioning illegal and dangerous ruck cleanouts in the 2018 Super Rugby competition with some dangerous illegal rucks not sanctioned by the referees (i.e., shoulder charge, neck roll, and contact above the shoulder). For coaches, Tozetto et al. analyzed the importance of coach turnover on team's performance in the Brazilian professional soccer league (2012-2017). Their results highlighted the impact of replacing the coach in terms of short-term performances and the minimal influence of the coach's prior experience. The second study by Bateman and Jones highlighted the importance of coach and performance analyst relationships in professional soccer. Their study used the COMPASS (conflict, openness, motivation, preventative strategies, assurance, support, and social networks) framework and identified the importance of all aspects to maintain a positive coach-analyst relationship. These articles highlighted the practical applications of this special issue for stakeholders (i.e., players, coaches, referees, and analysts).

Finally, the use of new technologies was also addressed in this Research Topic with a data driven visual prototype reported by Benito Santos et al.. This software technology employed geospatial data and visualization techniques and allowed stakeholders to understand the collective behaviors of soccer teams during competitions and training.

In light of the positive engagement and great number of high-quality articles published, this special issue has made an important contribution to exemplify and stimulate the evolving research sub-discipline of performance analysis in sport. We look forward to the current articles supporting future research approaches that consider the complex nature, emerging techniques and “big data” associated with sport.

## Author Contributions

All authors listed have made a substantial, direct and intellectual contribution to the work, and approved it for publication.

## Conflict of Interest

The authors declare that the research was conducted in the absence of any commercial or financial relationships that could be construed as a potential conflict of interest.

## References

[B1] Bertollo M. Doppelmayr M. Robazza C. (2020). “Using brain technologies in practice,” in Handbook of Sport Psychology, eds G. Tenenbaum and R. C. Eklund (Hoboken, NJ: Wiley), 666–693.

[B2] Borms J. (2008). Directory of Sport Science: A Journey Through Time: The Changing Face of ICSSPE. Champaign, IL: Human Kinetics.

[B3] Glazier P. S. (2017). Towards a grand unified theory of sports performance. Hum. Mov. Sci. 56, 139–156. 10.1016/j.humov.2015.08.001 26725372

[B4] Hughes M. Franks I. (2007). The Essentials of Performance Analysis: An Introduction. London: Routledge.

[B5] O'Donoghue P. (2009). Research Methods for Sports Performance Analysis. London: Routledge.

[B6] Robertson P. S. (2020). Man & machine: adaptive tools for the contemporary performance analyst. J. Sports Sci. 38, 2118–2126. 10.1080/02640414.2020.1774143 32530736

[B7] Woods C. T. McKeown I. O'Sullivan M. Robertson S. Davids K. (2020). Theory to practice: Performance preparation models in contemporary high-level sport guided by an ecological dynamics framework. Sports Med. Open 6, 1–11. 10.1186/s40798-020-00268-5 32797290 PMC7427670

